# Association between life’s essential 8 and frailty status among cancer survivors in the United States: a cross-sectional analysis

**DOI:** 10.1186/s12889-024-18741-1

**Published:** 2024-05-10

**Authors:** Xiuxiu Qiu, Qidong Wu, Yiyi Zhang, Yingjie Zhu, Ming Yang, Li Tao

**Affiliations:** 1 Department of Oncology, Longhua Hospital, Shanghai University of Traditional Chinese Medicine, Shanghai, China; 2 Department of Intensive Care Unit, Longhua Hospital, Shanghai University of Traditional Chinese Medicine, Shanghai, China; 3 Department of GCP, Longhua Hospital, Shanghai University of Traditional Chinese Medicine, Shanghai, China

**Keywords:** Life’s essential 8, Cancer survivors, NHANES, Frailty, Cross-sectional study

## Abstract

**Background:**

Frailty not only affects disease survival but also impacts the long-term function and quality life of all adults diagnosed with and/or treated for cancer.The American Heart Association has introduced Life’s Essential 8 (LE8) as a novel metric for assessing cardiovascular health. Currently, LE8’s application in evaluating the frailty of cancer survivors remains unreported. This research seeks to explore the connection between LE8 scores and frailty levels in cancer survivors across the United States, thereby addressing a significant void in existing studies.

**Methods:**

This study analyzed data from cancer survivors enrolled in the National Health and Nutrition Examination Surveys (NHANES) spanning the years 2005 to 2018, providing a comprehensive dataset. Multivariable logistic regression models were used to examine the linkage between LE8 rankings and frailty condition in cancer survivors. Furthermore, the study delved deeper into this correlation using restricted cubic spline (RCS) curves and subgroup analyses.

**Results:**

In the fully adjusted model, an increased LE8 level was closely associated with a reduced odds ratio of frailty among cancer survivors, with an OR of 0.95 (95% CI: 0.94–0.96, *p* < 0.0001).This pattern persisted across different categorizations of LE8 into low, moderate, and high groups, demonstrating a consistent trend. The analysis revealed a non-linear relationship between LE8 scores and frailty status, further supporting a straightforward association (*p*-value for non-linearity = 0.0729).

**Conclusion:**

Studies have found that the higher the LE8 score, the less likely a cancer patient is to develop debilitating symptoms.This indicates that the LE8 scores may provide an opportunity for interventions aimed at improving the prognosis of cancer patients.

## Introduction

The worldwide population of cancer survivors is steadily increasing, owing to the advancements of cancer treatment strategies. In the United States, the number of cancer survivors is projected to reach 26 million by 2040 [1]. This positive trend introduces new challenges, particularly in effectively managing and improving survivors’ long-term health. Foremost, among these challenges is frailty, a critical factor that profoundly impacts the quality of life of cancer survivors. Manifested as a clinical syndrome, frailty is characterized by persistent fatigue, muscle weakness, reduced mobility, and a compromised quality of life. Beyond impairing quality of life, frailty can heighten mortality risk and escalate healthcare resource utilization. Defined by diminished reserve and resilience against stressors, or as an aggregation of conditions leading to vulnerability [2], frailty renders cancer survivors especially susceptible to stressful events. These events include infections, chemotherapy, radiation, or surgery, further exacerbating their reduced capacity to withstand stressors.

Frailty may potentially be mitigated or even reversed through appropriate nutritional supplementation and regular physical activity, underscoring its significance as a pivotal intervention target. The American Heart Association’s “Life’s Essential 8” health metrics emerge as a key resource in assessing and promoting well-being, with particular focus on cardiovascular health [3]. These metrics encompass critical aspects including diet, physical activity, non-smoking, maintaining a healthy weight, cholesterol management, blood pressure regulation, blood sugar monitoring, and ensuring quality sleep [4], offering a comprehensive framework for health improvement.

While these indicators primarily target cardiovascular health, they are also relevant for improving overall health and addressing frailty in cancer survivors. Preliminary evidence indicates a strong association between lifestyle factors and the health status of cancer survivors.However, a targeted exploration of the relationship between the Life’s Essential 8 metrics and frailty among cancer survivors remains scarce. This research aims to bridge this gap by investigating the correlation between the Life’s Essential 8 metrics and frailty among cancer survivors. The objective is to provide innovative insights and strategies for the sustained health management and lifestyle interventions of cancer survivors.

## Materials and methods

### Data sources

NHANES (National Health and Nutrition Examination Survey) operates as a continual, nationally representative cross-sectional study in the U.S, employing a stratified multistage probability sampling method.Conducted by the National Center for Health Statistics, this survey aims to precisely capture the health and nutritional status of the U.S. population. For individuals seeking a thorough insight, the NHANES website offers an extensive explanation of the design methodology and data collection techniques. Please visit http://www.cdc.gov/nchs/nhanes.htm for comprehensive information.

### Study design and population

**Fig. 1:**
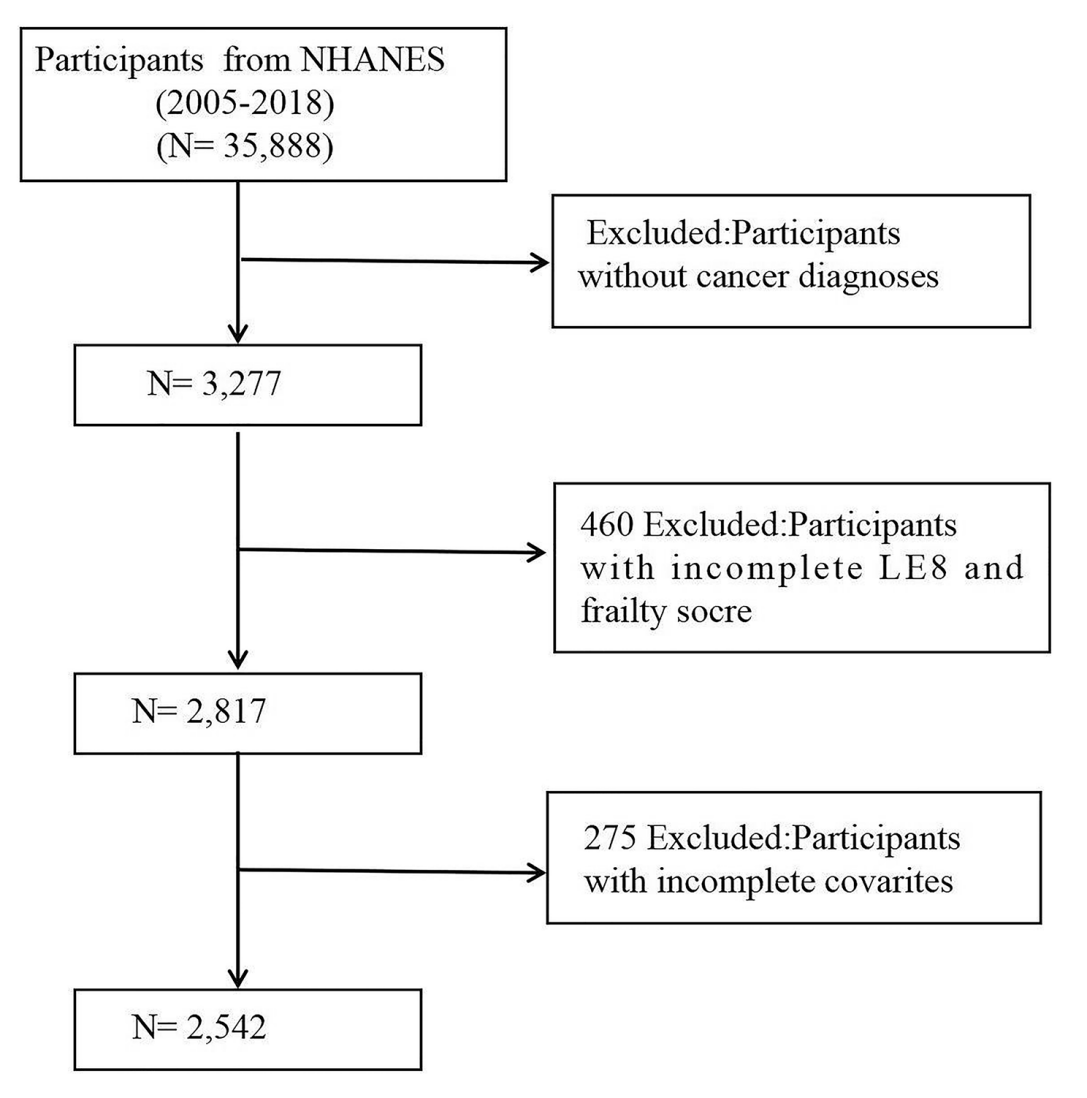
The flowchart in selecting the studying participants

The examination in this study analyzed information from 35,888 participants aged between 20 and 85 years, collected over seven cycles of the NHANES (National Health and Nutrition Examination Survey) spanning 2005 to 2018, with a concentration on individuals who have survived cancer. Initially, 3,277 participants were identified after being questioned if they had ever been informed by a doctor or another health professional about having cancer or any type of malignancy. Those who answered “Yes”were further queried about the type of cancer. Then, the selection criteria for tumor survivors were meticulously defined to ensure the integrity of the data: (1) excluding individuals under 18 years of age; (2) omitting participants who lacked complete scores for Life’s Essential 8 (LE8) and frailty; and (3) excluding those missing essential covariate data. Adhering to these standards, 2,542 participants were eventually included in the research (Fig. 1).

### Definition of life’s essential 8 (LE8)

The “Life’s Essential 8”(LE8) initiative by the American Heart Association is a critical advancement for enhancing cardiovascular health, encompassing eight essential elements: diet, physical activity, exposure to tobacco/nicotine, sleep quality, body mass index (BMI), levels of non-high-density lipoprotein cholesterol (non-HDL), blood glucose, and blood pressure. Within the LE8 framework, a key element for evaluating dietary patterns is the generation of the Dietary Approaches to Stop Hypertension (DASH) diet score, calculated from the average values of dietary components gathered through two nonconsecutive 24-hour dietary recalls at the outset [5].

Further enhancing the comprehensiveness of LE8’s assessment, standardized questionnaires meticulously collect additional data, encompassing self-reported minutes of moderate or vigorous physical activity per week, tobacco/nicotine exposure, sleep duration, and medication use. Physical measurements crucial to cardiovascular health—specifically, weight, height, blood glucose, and blood pressure—are accurately ascertained in Mobile Examination Centers (MEC) employing standard protocols. These measurements facilitate the calculation of BMI, a key indicator of cardiovascular risk, derived by dividing an individual’s weight in kilograms by the square of their height in meters.

The LE8 scores, as recommended by the American Heart Association, are stratified into three categories reflecting cardiovascular health status: low (LE8 < 50), moderate (50 ≤ LE8 < 80), and high (LE8 ≥ 80) [6]. Through such a rigorous methodology, the LE8 initiative provides a multifaceted approach to cardiovascular well-being, combining self-reported and objectively measured data to offer a comprehensive assessment of health factors pivotal to mitigating cardiovascular risk [7].

### Assessment of frailty

Frailty is considered to be the result of cumulative cellular damage, subsequently leading to a decline in organ system function and a reduced ability to restore homeostasis after stress events [8]. Searle et al.‘s standard procedure [9] is utilized to measure the frailty level across all study groups. The frailty index must incorporate health deficits as critical attributes. These attributes should be readily accessible, span various systems, and have a risk that escalates with age. The frailty index’s scale is from 0 to 1, where a higher score indicates increased frailty. A frailty index value greater than 0.21 defines frailty [10, 11]. The index comprises 53 variables [11], all present within the NHANES database. It encompasses cognition (1 item), dependency (20 items), depressive symptoms (7 items), comorbidities (13 items), utilization of hospitals and access to medical care (5 items), physical and anthropometric measurements (1 item), and laboratory data (6 items).

### Covariates

To minimize the impact of confounding factors on outcomes, we identified essential factors such as age, race/ethnicity, marital status, education level, economic status, body mass index (BMI), alcohol consumption, smoking history, and chronic conditions (including hypertension, diabetes, stroke, coronary heart disease, and various types of tumors) as major potential confounders. Gender was divided into two groups: male and female. Additionally, we segmented race/ethnicity into four categories: Non-Hispanic White, Non-Hispanic Black, Mexican American, and Other. Furthermore, educational levels were delineated into three categories: high school or above and below high school. Marital status was sorted into four classifications: “married or living with a partner,” “widowed,” “divorced or separated,” and “never married.”Body Mass Index (BMI) was calculated as weight (kg) divided by the square of height (m^2^) and classified into three groups: <25 kg/m^2^, 25–30 kg/m^2^, and > 30 kg/m^2^.Smoking status was categorized into three groups: never (those who have smoked fewer than 100 cigarettes in their lifetime), former (individuals who have smoked over 100 cigarettes in their lifetime but currently do not smoke), and current (people who have smoked more than 100 cigarettes in their lifetime and continue to smoke occasionally or daily).Alcohol consumption was divided into five categories based on the past year’s questionnaire responses: never (consumed less than 12 drinks in a lifetime), mild (≤ 2 drinks every day for males, ≤ 1 drink every day for females), moderate (≤ 3 and > 2 drinks every day for males, ≤ 2 and > 1 drink every day for females, or ≥ 2 to < 5 days per month of binge drinking), heavy (≥ 4 drinks every day for males, ≥ 3 drinks every day for females, or ≥ 5 days per month of binge drinking), and former (did not drink in the last year, but had ≥ 12 drinks in one year or has consumed ≥ 12 drinks in a lifetime). Chronic diseases were categorized as diabetes, hypertension, coronary heart disease (CHD), and stroke. The diabetes category was further broken down into diabetes mellitus (DM), compromised impaired fasting glycemia(IFG), and impaired glucose tolerance(IGT). The identification of these three conditions relied on self-reported data from patients. Cancer patients were additionally categorized into various types: breast cancer, digestive system cancers, gynecological, hematological, respiratory, skin or soft tissue, urinary system, and other tumor types.

### Statistical analysis

In our research, weighted methodologies were employed for statistical analysis. Categorical variables, expressed as percentages (%), underwent analysis through a weighted chi-square test. Continuous variables, presented with standard deviation (SD), were examined using a weighted t-tests. We categorized frailty into two segments: those without frailty (≤ 0.21) and those with frailty (> 0.21) [12]. Multivariable logistic regression models were employed to examine the relationship between LE8 scores and frailty status. Stratified analyses in different subgroups were conducted using logistic regression models. Furthermore, restricted cubic spline (RCS) analyses were performed. Interaction tests were used to determine the difference among various stratifications in the subgroups, with results displayed as Odds ratios (ORs). R 4.3.2 was carried out in this study. A two-sided *p* < 0.05 was deemed to indicate statistical significance.

## Results

### Baseline characteristics of study participants

Table 1 displays the initial characteristics of individuals who have survived cancer, segmented by their frailty status. Following the defined exclusion criteria, the study enrolled 2,542 cancer survivors, equivalent to an estimated 12,945,571 individuals in the United States. The mean age of these participants was 62.58 ± 0.38 years, with 57.34% identifying as female. Based on the frailty index utilized in this research, 33.74% were classified as “frail”.

**Table 1:** Characteristics of participants classified by frailty status

Variables	Overall	Without frailty	With frailty	*P*-value
		*N* = 1,504	*N* = 1,038	
**Age, (years)**	62.58 ± 0.38	61.25 ± 0.52	65.20 ± 0.50	< 0.0001
**Gender,%**				0.08
Male	1209(42.66)	746(44.16)	463(39.73)	
Female	1333(57.34)	758(55.84)	575(60.27)	
**Ethnicity/Race,%**				< 0.001
Mexican American	149(2.02)	77(1.73)	72(2.59)	
Non-Hispanic White	1789(87.78)	1095(89.60)	694(84.20)	
Non-Hispanic Black	353(4.87)	191(3.89)	162(6.80)	
Other	251(5.33)	141(4.78)	110(6.42)	
**Education level,%**				< 0.0001
High school or above	2336(95.99)	1430(97.77)	906(92.48)	
Below high school	206(4.01)	74(2.23)	132(7.52)	
**PIR**	3.36 ± 0.05	3.72 ± 0.05	2.67 ± 0.08	< 0.0001
**LE8**	65.59 ± 0.39	69.63 ± 0.45	57.66 ± 0.57	< 0.0001
**BMI**				< 0.0001
< 25	701(28.66)	480(33.01)	221(20.11)	
25–30	891(34.31)	539(34.91)	352(33.15)	
> 30	950(37.03)	485(32.08)	465(46.74)	
**Marital status,%**				< 0.0001
Married or living with a partner	1562(66.75)	1005(71.34)	557(57.73)	
Widowed	415(13.18)	202(10.30)	213(18.84)	
Divorced or separated	416(15.05)	202(12.73)	214(19.60)	
Never married	149(5.02)	95(5.63)	54(3.83)	
**Alcohol user, %**				< 0.0001
Former	577(17.74)	269(13.62)	308(25.83)	
Heavy	233(10.62)	132(10.50)	101(10.86)	
Mild	1107(46.95)	722(50.18)	385(40.62)	
Moderate	308(15.25)	209(17.53)	99(10.78)	
Never	317(9.44)	172(8.18)	145(11.91)	
**Smoking status,%**				< 0.0001
Former	1034(39.13)	609(38.61)	425(40.17)	
Never	1135(45.84)	718(49.00)	417(39.64)	
Now	373(15.02)	177(12.39)	196(20.19)	
**Hypertension,%**				< 0.0001
No	915(42.42)	688(51.59)	227(24.41)	
Yes	1627(57.58)	816(48.41)	811(75.59)	
**Diabetes,%**				< 0.0001
DM	680(22.37)	256(14.86)	424(37.11)	
IFG	131(5.49)	89(5.94)	42(4.61)	
IGT	134(4.51)	95(5.10)	39(3.34)	
No	1597(67.63)	1064(74.10)	533(54.94)	
**Stroke,%**				< 0.0001
No	2317(93.69)	1455(98.02)	862(85.38)	
Yes	222(6.24)	48(1.98)	174(14.62)	
**CHD,%**				< 0.0001
No	2295(92.31)	1443(96.97)	852(83.16)	
Yes	247(7.69)	61(3.03)	186(16.84)	
**Tumor type,%**				< 0.0001
Breast	402(15.69)	238(15.65)	164(15.77)	
Digestive system	206(6.05)	94(4.65)	112(8.81)	
Gynecological	318(12.92)	164(11.85)	154(15.02)	
Hematological	96(3.81)	55(3.89)	41(3.65)	
Respiratory	63(2.09)	28(1.50)	35(3.24)	
Skin or soft tissue	761(38.09)	508(41.86)	253(30.68)	
Urinary system	401(10.45)	262(10.57)	139(10.22)	
Other	295(10.91)	155(10.03)	140(12.62)	

Statistical analyses identified significant differences across several variables between the frail and non-frail groups, including age, race/ethnicity, education level, smoking status, alcohol consumption, poverty-income ratio (PIR), Life’s Essential 8 (LE8) score, Body Mass Index (BMI), and prevalence of hypertension, diabetes, stroke, coronary heart disease (CHD), and tumor type (all *p* < 0.05). However, no significant disparities were found regarding gender.

### Association between LE8 scores and Frailty Status

The investigation into the correlation between the Life’s Essential 8 (LE8) score and frailty among cancer survivors employed three distinct logistic regression models.Model I made adjustments for age, ethnicity, and gender. Model II built upon Model I, incorporating adjustments for marital status, Body Mass Index (BMI), educational level, alcohol user, smoking habits, and the poverty-income ratio (PIR). Model III extended these adjustments to include hypertension, diabetes mellitus (DM), stroke, coronary heart disease (CHD), and various tumor types. When LE8 treated as a continuous variable, showed that each unit increase in the LE8 score was associated with a 6% reduction in the odds of frailty in both Model I and Model II (OR = 0.94, 95% CI [0.93, 0.95],*p* < 0.0001 for both models); Model III continued to substantiate the LE8 score’s protective effect with an even greater reduction in frailty odds (OR = 0.93, 95% CI [0.92, 0.94], *p* < 0.0001), underscoring the potential of lifestyle modification in mitigating frailty risk. (refer to Table 2).

**Table 2:** Odds ratios and 95% confidence intervals for the association between LE8 and frailty status

Exposure	Model I^a^ (OR,95%CI,*P*-value)	Model II^b^ (OR, 95% CI, *P*-value)	Model III^c^ (OR, 95% CI, *P*-value)
LE8 score	0.94(0.93,0.95) < 0.0001	0.94(0.93,0.95) < 0.0001	0.95(0.94, 0.96) < 0.0001
**LE8 score, (groups)**			
Low (LE8 < 50)	ref	ref	ref
Moderate (50 ≤ LE8 < 80)	0.25(0.18,0.34)< 0.0001	0.36(0.25,0.52)< 0.0001	0.45(0.30, 0.68)< 0.001
High (LE8 ≥ 80)	0.06(0.04,0.10)< 0.0001	0.14(0.08,0.23)< 0.0001	0.22(0.13, 0.40)< 0.0001

^a^Model I included adjustments for age, ethnicity, and gender

^b^Model II incorporated adjustments for age, ethnicity, gender, marital status, Body Mass Index (BMI), educational level, alcohol consumption, smoking status, and the poverty-income ratio (PIR)

^c^Model III included adjustments for age, ethnicity, gender, marital status, Body Mass Index (BMI), educational level, alcohol consumption, smoking status, poverty-income ratio (PIR), hypertension, diabetes, stroke, coronary heart disease(CHD), and tumor types

The trends persisted when the analysis was conducted with the LE8 score as a categorical variable. In Model I, the high LE8 score group (LE8 ≥ 80) showed a significantly reduced odds ratio (OR) for frailty (0.06, 95% CI [0.04, 0.10], *p* < 0.0001) compared to the low LE8 score group (LE8 < 50). The fully adjusted Model (Model III) also indicated a significantly stronger association between a high LE8 score and reduced frailty odds, underscoring a clear trend (*p* for trend < 0.0001).The stark contrast in frailty odds between the high and low LE8 score groups underscores the critical role of comprehensive lifestyle interventions in mitigating frailty risk.

### Identifying Non-linear relationships among study variables

**Fig. 2:**
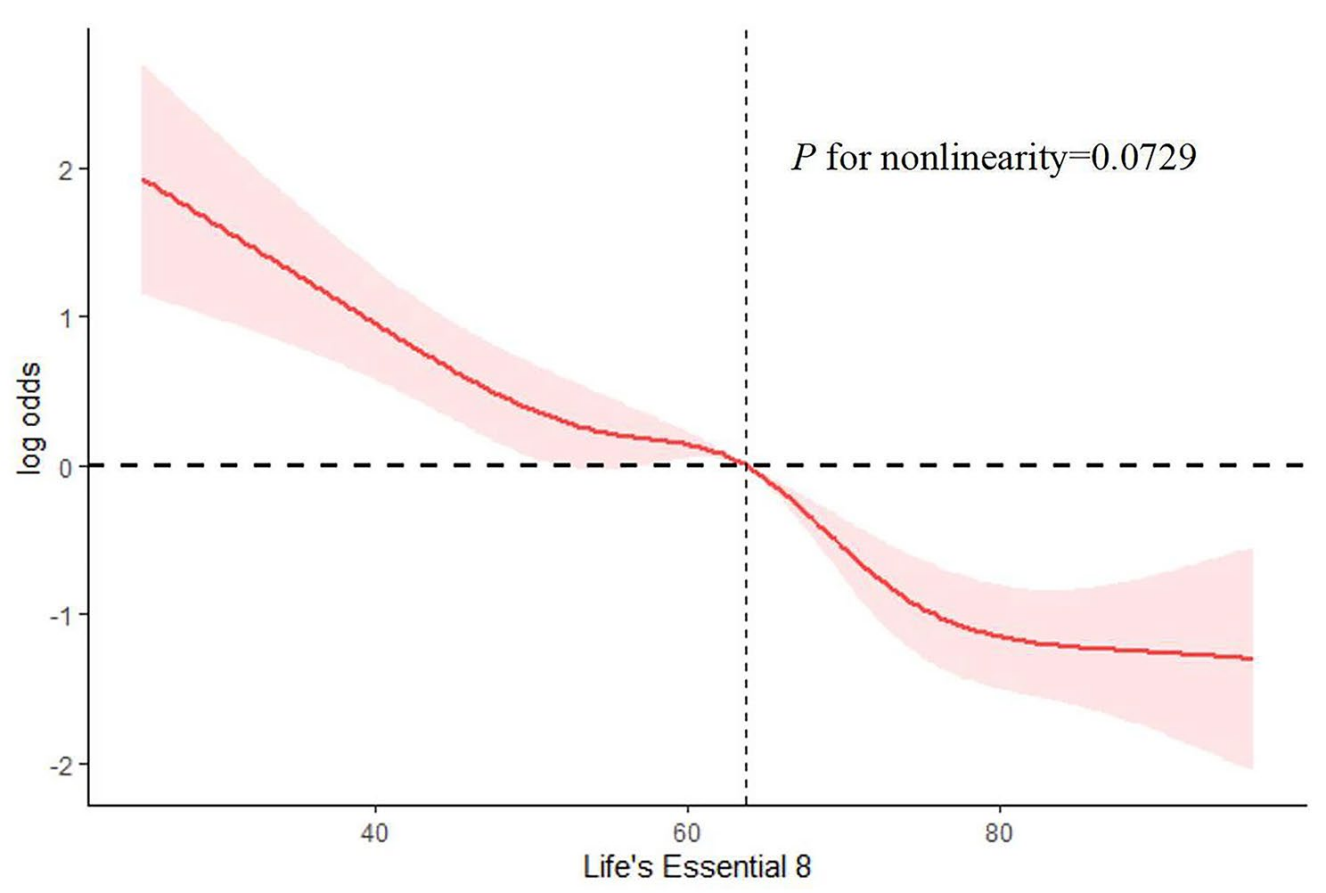
The RCS curve illustrates the association between Life’s Essential 8 and frailty status among all cancer survivors. RCS regression was adjusted for age, ethnicity, gender, marital status, Body Mass Index (BMI), educational level, alcohol consumption, smoking status, poverty-income ratio (PIR), hypertension, diabetes, stroke, coronary heart disease (CHD), and tumor types

To elucidate the nature of the relationship between the Life’s Essential 8 (LE8) score and the incidence of frailty in cancer patients, a sophisticated analytical method was employed, utilizing a restricted cubic spline regression model to represent the approach (Fig. 2). The results indicated there was no significant non-linear relationship between the LE8 score and frailty (*p* for non-linearity = 0.0729).This outcome indicates that, within the range of data analyzed, the relationship does not significantly deviate from linearity, suggesting that the association between the LE8 score and frailty risk may be more straightforward than previously anticipated.

### Subgroup analysis stratified by clinically important covariates

**Fig. 3:**
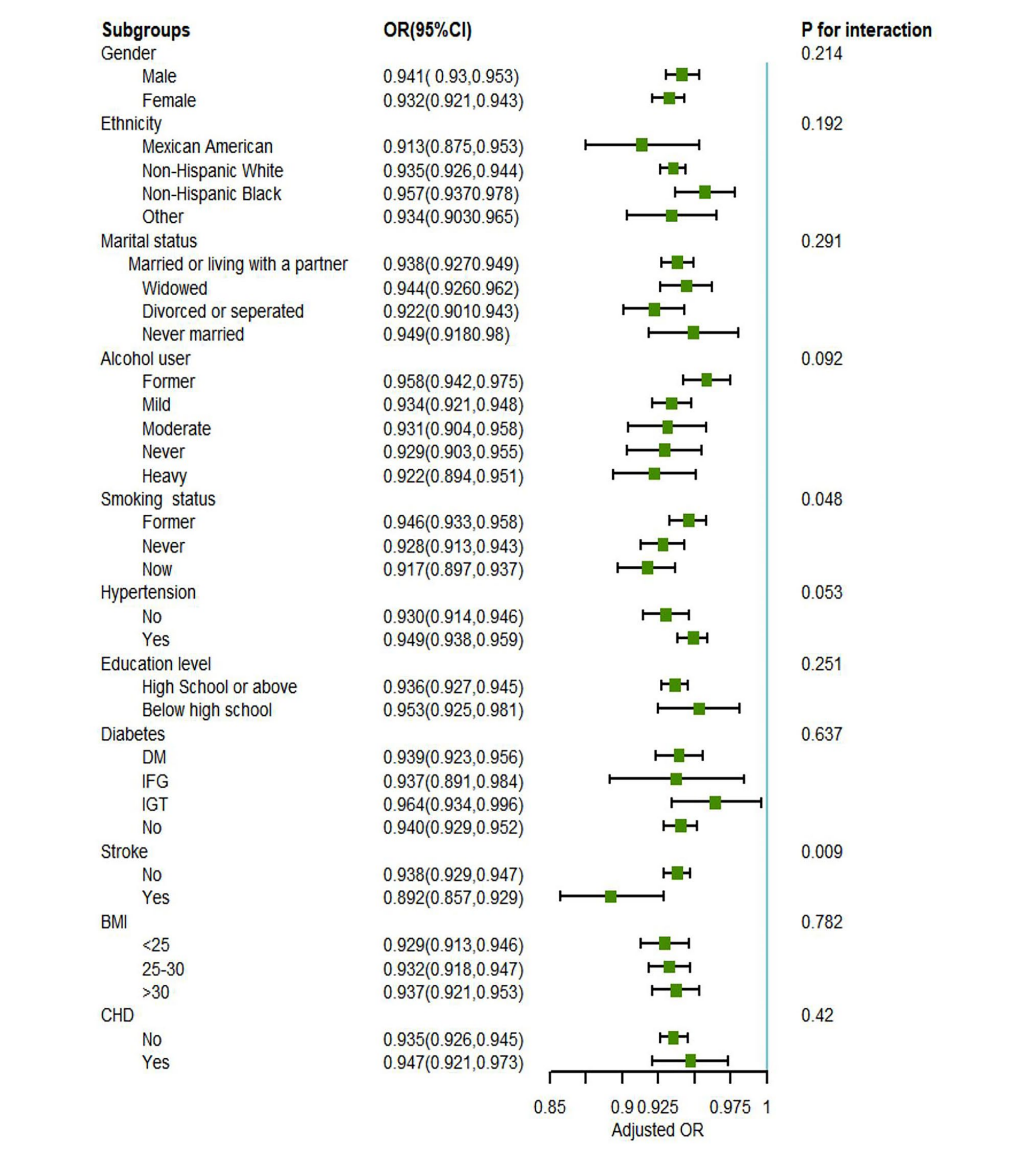
Subgroup analysis explored the association between Life’s Essential 8 scores and frailty status. Each stratification was adjusted for gender, ethnicity, marital status, alcohol consumption, smoking status, hypertension, diabetes, stroke, Body Mass Index (BMI), and coronary heart disease (CHD)

Upon adjusting for pertinent covariates, the study proceeded to examine the interactions and carry out subgroup analyses concerning Life’s Essential 8 (LE8) score and frailty. The subgroup analyses were conducted, stratifying by factors including gender, race/ethnicity, level of education, marital status, Body Mass Index (BMI), smoking behavior, alcohol intake, hypertension, diabetes, and coronary heart disease (CHD), revealed no significant interactions, indicating the relationship between the LE8 score and frailty levels was consistent across these variables (all *p*-values for interactions > 0.05). This uniformity underscores the robustness of the LE8 score as a reliable indicator of frailty risk, unaffected by the wide range of demographic and health-related factors considered in this study.However, a notable exception was observed regarding stroke, where a significant effect modification in the relationship between the LE8 score and frailty status was detected (*p* for interaction < 0.05), highlighting the unique influence of stroke in this association (Fig. 3).

## Discussion

Frailty, a syndrome characterized by diminished strength, endurance, and reduced physiological capacity, significantly increases vulnerability to various stressors, including cancer, chemotherapy, radiotherapy, and surgical interventions [13, 14]. Recognizing the pronounced susceptibility of cancer survivors to frailty, particularly due to age-related decline and treatment side effects [15], this study aimed to delineate the relationship between cardiovascular health, as measured by the Life’s Essential 8 (LE8) metrics, and the occurrence of frailty in this population.

Utilizing a cross-sectional approach, our study examined data from 2,542 individuals who participated in the National Health and Nutrition Examination Survey (NHANES) from 2005 to 2018. This comprehensive analysis revealed a notable linear dose-response relationship between the Life’s Essential 8 (LE8) scores and frailty, indicating that higher cardiovascular health levels correspond to a reduced frailty risk among cancer survivors. Particularly striking was the discovery of a significant interaction within the stroke subgroup during the analyses, which highlights the nuanced complexity of these associations.

Cancer survivors, already burdened by their past illness, face a heightened risk of frailty—a condition associated with a spectrum of adverse health outcomes [1, 16]. Our findings highlight the potential of interventions focused on enhancing cardiovascular health, as measured by Life’s Essential 8 (LE8) scores, to mitigate the risk of frailty in this vulnerable population. These results suggest that integrating strategies to improve cardiovascular health into the comprehensive care of cancer survivors could significantly enhance their quality of life and long-term well-being.

Building on this, our well-designed study and rigorous analysis identified a strong association between optimal cardiovascular health and a significantly reduced risk of frailty among the cancer population. Additionally, it’s important to note that cancer survivors may become susceptible to cardiac tumors, potentially exacerbating their frailty. The occurrence of cardiac tumor reactions may increase the risk of debilitation, highlighting the critical need for cardiovascular monitoring in this group.

These findings not only reinforce the vital role of cardiovascular health in the recovery and long-term well-being of cancer survivors but also offer compelling justification for integrating cardiovascular health interventions into cancer recovery programs. Unlike previous research, which predominantly examined the relationship between cardiovascular health and functional status in the general population [15], our study highlights the critical importance of regular cardiovascular monitoring during and after cancer treatment in cardio-oncology. This monitoring facilitates the timely identification of potential cardiac complications arising from cancer treatments, such as cardiotoxic reactions. Early detection of these signs allows medical teams to implement interventions promptly, thereby protecting the patient’s cardiac health and improving their quality of life.

The results emphatically support the necessity of a holistic approach to post-cancer care that includes comprehensive management of cardiovascular health as a strategy to mitigate and prevent frailty. Notably, our analyses have determined that among the Life’s Essential 8 (LE8) indicators, diet, physical activity, and sleep quality are the most influential factors in diminishing the symptoms of frailty. This observation aligns with existing literature, reinforcing the crucial role of lifestyle modifications in enhancing the quality of life for cancer survivors. Given these findings, cardiovascular interventions should be considered an integral part of the cancer recovery process, particularly for those patients at risk of cardiac health issues due to their treatment. To further support this, we advocate for targeted health interventions that promote dietary modifications, encourage regular physical activity, and enhance sleep quality as foundational elements of cancer survivorship care plans.

However, this study is subject to several limitations. Firstly, the inherent nature of the cross-sectional design limits our ability to infer causality between improvements in cardiovascular health and changes in frailty status among cancer survivors. This underscores the need for longitudinal research to more definitively explore the dynamics of this relationship. Secondly, despite thorough efforts to account for a broad spectrum of potential confounders, we must acknowledge the possibility that other, unmeasured variables may influence our findings. Additionally, given that our sample was sourced from a specific region and demographic, caution is advised when extrapolating these results to a broader population.

Future research should focus on several key areas. Firstly, establishing a longitudinal framework is crucial to elucidate the causal connections between cardiovascular health metrics and frailty among cancer survivors. Secondly, it is imperative to investigate the impact of cardiovascular interventions on frailty risk, particularly within the cardio-oncology patient population. This should include assessing the efficacy of various cardiovascular interventions, such as exercise, dietary adjustments, and pharmacotherapy, in preventing or mitigating frailty. Furthermore, to enhance the robustness and wider applicability of these findings, it is essential to expand the scope of research to include a diverse array of populations and geographic settings. Such efforts will not only validate the generalizability of our results but also deepen our understanding of the complex relationship between cardiovascular health and frailty in cancer survivors.

## Conclusion

In conclusion, this investigation highlights the crucial role of cardiovascular health in mitigating frailty among cancer survivors. By focusing on optimizing the Life’s Essential 8 cardiovascular health metrics, our findings suggest a promising avenue for enhancing health management in this population. This approach is expected not only to improve their overall quality of life but also to positively impact long-term health outcomes. Thus, this study contributes valuable insights into the interdisciplinary fields of oncology and cardiovascular health, underscoring the importance of integrated health strategies in supporting the well-being of cancer survivors.

## Data Availability

The database for NHANES (https://www.cdc.gov/nchs/nhanes/index.htm) houses all accessible data.

## Abbreviations

LE8: Life’s Essential 8
CHD: Coronary heart disease
NHANES: National Health and Nutrition Examination Survey
IFG: Impaired fasting glucose
IGT: Impaired glucose tolerance
DM: Diabetes mellitus
BMI: Body Mass Index
OR: Odds Ratio
CI: Confidence Interval
P-value: In bold indicates statistical significance
